# A Pilot Study of a Group Program Focused on Enabling Life Performance for Older Adults Living in the Community

**DOI:** 10.3390/ijerph19073761

**Published:** 2022-03-22

**Authors:** Kenichiro Furuta, Norikazu Kobayashi, Ryuji Kobayashi, Hitomi Ishibashi, Yu Ishibashi

**Affiliations:** 1 Department of Occupational Therapy, Graduate School of Human Health Sciences, Tokyo Metropolitan University, Tokyo 116-8551, Japan; yuikyuik0326@gmail.com (K.F.); n-koba@tmu.ac.jp (N.K.); ryukoba@tmu.ac.jp (R.K.); 2 Department of Occupational Therapy, Faculty of Medical and Health Sciences, Tokyo University of Technology, Tokyo 144-8535, Japan; hishibashi@stf.teu.ac.jp

**Keywords:** activity and participation, community life support, care prevention

## Abstract

Background: This study attempts to determine whether a program focused on improving literacy in daily living is effective in preventing physical frailty, and to compare standard treatments for physical frailty. Methods: This study was designed as a pilot intervention study involving two groups. Twenty-five older adults aged 65 to 85 in Ward A, Tokyo, were randomly assigned to the literacy group or the exercise group on a regional basis and were given a 60- to 90-minute program twice a month, eight times over four months. The literacy group mainly used video materials to monitor learning, and the exercise group used a multifactor exercise program. Results: The LSI-Z, GAS-L, Maximum 5 m walking time, and TUG tests showed the main effects before and after the intervention in both groups (*p* < 0.05, *p* < 0.01). The WHOQOL26, Maximum 5 m walking time, and TUG tests also showed the main effects across both groups (*p* < 0.05). Conclusion: Both programs, when implemented independently, showed specific effects on subjective well-being, occupational performance, and physical fitness. However, QOL and physical fitness were significantly higher in the exercise group than in the literacy group. These results should be considered with caution because of the limited sample size of this pilot study.

## 1. Introduction

In recent years, frailty in older adults has been increasingly studied worldwide. According to Fried, frailty has been examined from three aspects: physical, mental, and social [1]. Research on frailty is growing, and early detection and prevention efforts are being established. Satake et al. developed a Japanese version of the Cardiovascular Health Study (CHS) criteria for assessing physical frailty [2]. In addition, Yamada et al. and Makizako et al. reported a method to identify socially frail older adults using a simple screening test [3,4]. A systematic review by Yoshimura et al. found that improving walking and muscle strength was effective in improving physical frailty [5]. Other studies have examined the effects of programs that combine nutritional guidance and other factors [6,7]. Thus, preventing frailty in older adults is an important initiative for extending healthy life expectancy.

One of the objectives of frailty prevention is to prevent disabilities in daily life. Life disability in older adults is mainly caused by instrumental activities of daily living (IADL), and is reported as a narrowing of the range of daily activities [8]. There have been reports of support for life disabilities, focusing on literacy in daily life [9]. However, these reports have not been examined from the perspective of frailty prevention, and it is unclear whether support focusing on daily life disabilities effectively prevents physical frailty. Similarly, it has not been verified whether a program focusing on physical frailty effectively improves life disability. In this study, we considered that it is necessary to examine whether a program for the prevention of physical frailty affects the maintenance and improvement of daily life tasks, and whether a program for the prevention of life disabilities has an effect on the maintenance and improvement of daily life tasks and the prevention of physical frailty.

This study aimed to determine whether a program focused on improving literacy in daily living effectively prevents physical frailty, and compares standard treatments for physical frailty. We believe that the results of this study are a resource for characterizing both programs and examining how they contribute to physical frailty and life disability.

## 2. Materials and Methods

### 2.1. Research Design

We conducted a pilot study comparing the treatments (the Enabling Life Performance Program and the Physical and Mental Function Improvement Program) using a one-group pre-intervention versus post-intervention design. To avoid publication bias, we registered in advance with the UMIN Clinical Trials Registry (UMIN000032307).

### 2.2. Study Subjects, Recruitment Method, and Period

The study subjects were: (1) those aged 65–85 years who were not certified as requiring level-one nursing care or higher, and (2) older adults who could visit the program site by themselves or with a family member’s escort. The exclusion criteria were: (1) those who could not come to the program site, (2) those who could not understand the study’s content, and (3) those who were not willing to cooperate in the study. We searched local government websites for local comprehensive support centers, nursing care prevention promotions, and daycare services, calling for their cooperation in recruiting participants. We asked those who cooperated to post and distribute posters for recruitment of research subjects. In addition, we explained the study’s framework to the subjects and called for their participation. Subsequently, we held a research briefing session for those who agreed to participate and gave their consent. The recruitment period was 1 April–21 June 2018, and the recruitment of subjects began with the cooperating centers and services. The program was implemented from 25 May 2018 to 13 October 2018.

### 2.3. Allocation and Blinding

Ward A of Tokyo was geographically divided into seven districts, and this district division was used as one unit of the cluster. Random numbers were generated using Microsoft Excel for random cluster assignment, and assignments to the literacy and exercise groups were block-randomized (block size 4). The actual allocation was performed by an OT who was not involved in the program.

### 2.4. Overview of the Program

#### 2.4.1. A Framework of the Program

The first author conducted all interventions. The maximum number of participants per program was approximately 15, and each program lasted 60 to 90 min for about four months, for a total of eight sessions (Table 1). In the first session, the participants were asked to clarify the life performance goals they wanted to achieve, and to evaluate their progress (Table 1). Programs for the literacy and exercise groups were conducted from the second to seventh sessions. In the fifth session (mid-program), we reviewed participants’ progress toward achieving their life goals. We then asked them to improve their life performance. In the eighth session, the degree of achievement of daily life goals was confirmed, and each evaluation conducted in the first session was conducted again. We did not pre-test the suitability of each program in this study. However, the literacy program was designed based on the same subject’s previous studies, and the exercise program was based on the Ministry of Health, Labour and Welfare (MHLW)’s program. The details of each program will be discussed later.

#### 2.4.2. Literacy Group

The newly developed Enabling Life Performance Program was implemented in the literacy group. The program is a literacy program that aims to set goals based on the lifestyle and values of the participants in order to improve their life disabilities, and, through lectures and group work sessions, help them reaffirm what they want to do and reflect their life skills in their own goals. The goal-setting method was based on the life goal-setting technique of Yuri et al., and goals were set by asking the participants how well they were performing their life tasks and how satisfied they were with their performance [10]. In addition, lectures and group work were structured for the same amount of time in one session each, referring to the health-promotion program by Kawamata et al. and the makeup program based on social participation support by Ishibashi et al. [11,12].

**Figure 1:**
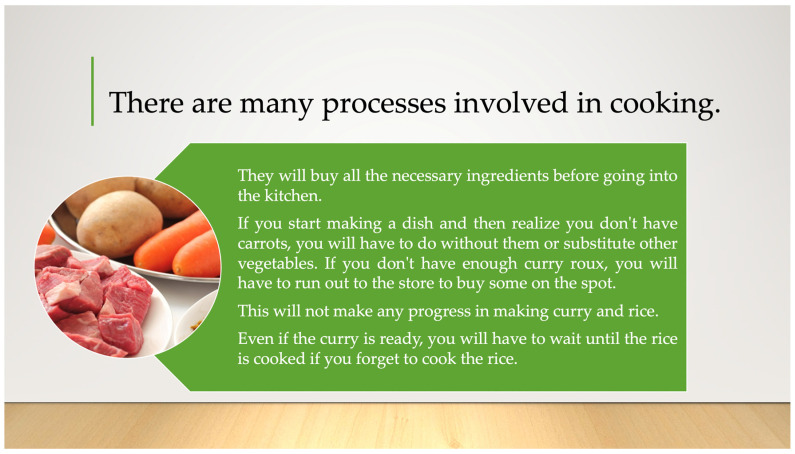
Excerpts from actual lecture materials.

Participants learned the importance of daily routines and adapting to life with compensatory means in the lectures, and devised effective ways to use their time. The content of the lectures was assembled regarding the Ottawa Charter for Health Promotion [13], and the Occupational Therapy Intervention Process Model [14], and mainly focused on quality of life, life and health, organization of space and objects, an adaptation of temporal organization and performance, and occupational analysis (Figure 1). Group work was developed based on observational learning (modeling), a component of Bandura’s social learning theory. Bandura proposes that one can learn by observing a model’s behavior, even though the model (exemplar) does not perform the behavior and no reinforcement is given [15]. The literacy group was asked to observe a simulated patient’s life scene played by a healthy adult, and to reflect on daily life tasks and how they could be made safer and performed more comfortably. For this program, we prepared four videos (moving furniture and vacuuming, preparing tea, making toast and eggs, cleaning a grave) and asked participants to observe scenes of the simulated patients’ daily lives and examine the related problems and issues. During the program period, participants were encouraged to apply the contents learned in each program to their daily lives as homework.

#### 2.4.3. Exercise Group

The physical and mental function improvement program was conducted according to the care prevention manual [16]. The goal-setting method used in this program was the same as that used for the literacy group. The contents and time allocation of the program were determined and implemented by referring to the manual; the goal was to improve locomotor functions, thus preventing the need for nursing care. Each program session focused on maintaining and improving mobility functions—the basis of daily life—through gymnastics exercises to prevent knee pain, back pain, falls and fractures, and on study time.

Gymnastics sessions included a warm-up, main exercise, and cool-down exercises. Before participating in gymnastics, participants were asked to reflect on their exercise habits at home; afterwards, they were asked to decide on the exercises they would perform at home before the next session as homework.

### 2.5. Measurements

#### 2.5.1. Demographics

Demographics were obtained using a self-administered questionnaire regarding gender, age, certification regarding requiring long-term care, the presence or absence of disease, family structure, frequency of outings, and the presence or absence of habits such as walking or exercising. Responses regarding certification for requiring long-term care were “not applicable”, “Support 1”, or “Support 2”. Respondents were also asked whether they had any diseases. Responses regarding family structure included “husband or wife”, “parents”, “siblings”, “son or daughter”, “daughter-in-law or son-in-law”, “grandchildren”, “lives alone”, and “other”. Responses for the frequency of going out included “almost every day”, “four to five times per week”, “two to three times per week”, “less than once a week”, and “rarely”. Participants were asked whether they were walking or exercising.

#### 2.5.2. Outcomes

The selection of outcome measures was based on the MHLW policy and previous studies, including the Goal Attainment Scaling-Light (GAS-L) to assess the degree of achievement of lifestyle goals, the WHO/QOL26 for assessing Quality of Life (QOL), the TMIG Index of Competence (TMIG-IC) to assess functional capacities for living, and the Life Satisfaction Index (LSI-Z) to assess life satisfaction. Other measures included grip strength, maximum 5 m walking time, and the Timed Up and Go (TUG) test to measure physical fitness [17,18,19,20,21,22].

The GAS-L is a shortened version of the GAS. The latter is an evaluation method that assesses a subject’s degree of achievement after an actual intervention, based on the predicted results. First, the tasks to be addressed by each subject were established. Next, we set five goal-achievement levels for each subject, and determined the desired outcomes in advance. We used the crude score obtained from the GAS-L final assessment: 1 for baseline, 0 for the expected outcome, and +1 for a different outcome.

The WHOQOL26 is a validated and reliable questionnaire that measures subjective well-being and QOL. It consists of 26 items: 24 items for QOL in four domains (physical, psychological, social, and environmental) and 2 items for overall QOL.

The higher the TMIG-IC score, the greater the ability to maintain a social life (range: 0–13). The TMIG-IC can calculate the ability to perform activities for an active daily life, engage in active intellectual activities (e.g., leisure and creative activities), and have a social role in the community, with subscale scores ranging from 0 to 5 and 0 to 4.

The LSI-Z assesses “enjoyment of daily life”, “acceptance of life as meaningful and present”, “the realization of the achievement of major goals”, “having a positive self-image”, and “maintaining a happy and optimistic mood”. Responses determine whether an individual can affirm that they maintain a sense of psychological well-being. The procedure begins with the subject filling out an evaluation form. Positive responses receive 2 points, and negative reactions receive 0 points, for a maximum total score of 26 points.

The physical fitness tests used were grip strength (evaluating muscle strength), maximum 5 m walking time (evaluating walking ability), and the TUG test for evaluating complex movement ability. These tests were conducted twice, and the best values were taken. Grip strength was measured twice for each hand, and the average of the best values was used. For 5m walking time and TUG, the smaller the value, the higher the ability.

### 2.6. Sample Size

Since this was a pilot study, the purpose of this study was also to estimate future sample sizes by establishing an estimate of the effectiveness of the intervention. Therefore, we recruited a maximum of 60 participants while referring to similar previous studies.

### 2.7. Statistical Analysis

For each outcome measure, a split-plot analysis of variance with two factors was applied: literacy group and exercise group (factors without correspondence), and before-and-after intervention (factors with correspondence). MacOS version(Apple, Cupertino, CA, USA) R Commander 2.7 (Mcmaster University, Hamilton, ON, Canada)–0 (R4.0.2; CRAN, freeware) was used for data analysis, and the significance level was set at less than 5%. All statistical analyses were conducted using the second author, masked.

### 2.8. Ethical Considerations

We respected the privacy of the subjects. The request for cooperation was made after the research was briefed. At the end of the briefing session, there was an opportunity to ask questions, and participants were informed that they could discontinue their participation if they felt burdened during the study. They were also advised that there would be no disadvantage incurred from discontinuing participation. This study was approved by the Research Safety Ethics Committee of the Tokyo Metropolitan University Arakawa Campus (Approval No. 17106) and was performed in accordance with the Declaration of Helsinki.

## 3. Results

### 3.1. Analysis Subjects and Follow-Up Rates

**Figure 2:**
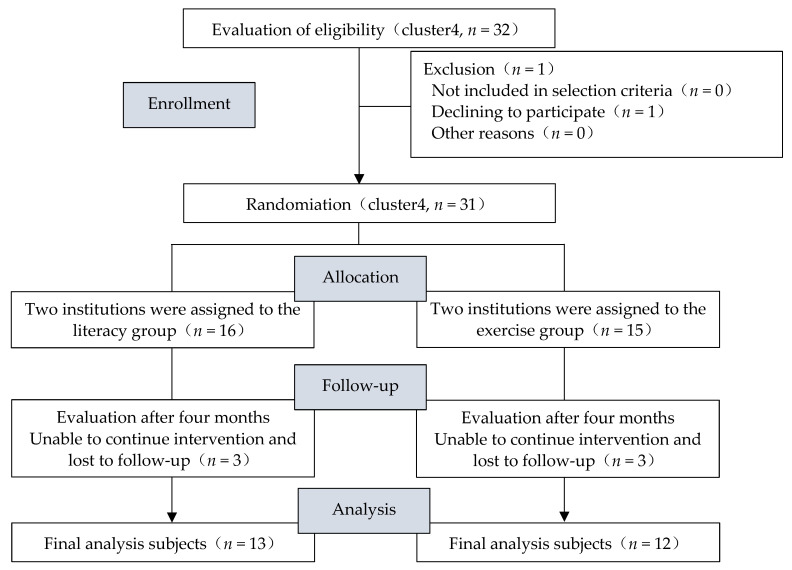
Flowchart showing cluster random assignment and the process of each stage.

Four institutions responded to recruitment efforts, and 32 were recruited (Figure 2). The subjects were assigned to the literacy or exercise group according to the prearranged allocation method. As a result, 16 participants were assigned to the literacy group and 15 to the exercise group (one participant withdrew). There were three untraceable participants in both the literacy and exercise groups. The follow-up rate (number of subjects analyzed/number of subjects at the time of assignment) was 81.3% in the literacy group and 80.0% in the exercise group.

### 3.2. Demographics and the Average Number of Programs Attended

In the final analysis, demographics were summarized by item (Table 2). The mean age ± standard deviation of those in the literacy group (*n* = 13; 3 males and 10 females) was 75.8 ± 4.76 years. The mean age ± standard deviation for the subjects in the exercise group (*n* = 12; 2 males and 10 females) was 76.5 ± 5.5 years. The mean number of programs attended was 7.1% (89.4%) for those in the literacy group and 7.2 (90.6%) in the exercise group.

### 3.3. Comparison of Two Factors for Outcome Measures

A comparison of the two factors on outcome measures is shown in Table 3. The LSI-Z, GAS-L, Maximum 5 m walking time, and TUG tests showed the main effects in both groups before and after the intervention (*p* < 0.05, *p* < 0.01). The WHOQOL26, Maximum 5 m walking time, and TUG tests also showed the main effects across both groups (*p* < 0.05). No interaction was found for any of the outcome measures.

## 4. Discussion

### 4.1. Program Feasibility

The study subjects’ follow-up rates were 81.3% in the literacy group and 80.0% in the exercise group. Three subjects were not followed up in each of the two programs. The average attendance rates for each program, excluding those with no follow-up, were 89.4% in the literacy group and 90.6% in the exercise group. According to the ACP Journal Club, the follow-up rate for randomized controlled trials should be at least 80%, and this study met this requirement, indicating the feasibility of the program [23].

### 4.2. Effects of the Program

Both groups showed better improvement in LSI-Z, GAS-L, Maximum 5 m walking time, and TUG tests after the intervention compared to before (*p* < 0.05, *p* < 0.01). In other words, when each program was conducted independently, a specific effect was observed on subjective well-being, occupational performance, and physical fitness. Kinoshita investigated subjective well-being and participation in activities among community-dwelling older adults and found a positive correlation for those aged 65–84 [24]. Berger conducted a systematic review of the occupational performance and quality of life of community-dwelling older adults and reported the need for a combined group and individual intervention to improve occupational performance [25]. In this study, both programs combined individual and group activities, with goals set at the individual level, followed by a group program to examine life performance issues and exercise. In addition, we believe that continuous participation in the program might lead to an increase in participants’ activity, contributing to their subjective well-being and improving their occupational performance.

In contrast, the WHOQOL26, Maximum 5 m walking time, and TUG test results were higher than the literacy group, before and after the intervention, in the exercise group (*p* < 0.05). Jie Zhuang reported that a 12-week multifactorial exercise program in a randomized controlled trial for community-dwelling older adults effectively improved physical fitness, including the TUG test [26]. In addition, Bouaziz et al. reported that a systematic review of the effects of a multifactorial exercise program in older adults aged 65 years and older showed positive outcomes on quality of life [27]. Although the small sample size makes it difficult to draw any general conclusions, we infer that the exercise group’s program results may support those of previous studies.

The results obtained in this study indicate that literacy programs might play a positive role in subjective well-being, occupational performance, and physical fitness. On the other hand, the exercise program showed more favorable results for quality of life and physical fitness than the literacy program. In other words, although the literacy program may be effective in preventing physical frailty, the standard of care may contribute more to improving quality of life and physical fitness.

### 4.3. Limitations and Future Prospects

We have conducted a pilot study comparing the two treatments (the Enabling Life Performance Program and the Physical and Mental Function Improvement Program) using one-group pre-intervention versus post-intervention design. This allows us to make suggestions for future studies with larger data sets. First, we were able to estimate the approximate sample size. When the ideal sample size was set at 80% power, 5% level of significance, and median effect size d = 0.50, and the outcome measure was analyzed with a *t*-test, the number of subjects required was calculated to be 64 for each group. Since approximately 20% of the subjects dropped out of the study, we believe that it is necessary to recruit more than 77 subjects and conduct intergroup comparisons using a control group in the future. Second is the issue of the outcomes. The TMIG-IC used in this study showed a ceiling effect from the baseline, and the amount of change before and after the intervention was small. Finally, there are limitations of validity due to the study design. In the present study, we compared the effectiveness of the programs using a one-group pre-intervention versus post-intervention design. However, the study design did not involve a control group, and it is undeniable that the lack of physical intervention for the literacy group may have affected the results. Although increasing the number of measurement points without changing the study design is one way to increase validity, we believe that randomized controlled trials with a higher level of evidence are needed.

## 5. Conclusions

The purpose of this study was to determine whether a program focusing on improving literacy in daily living is effective in preventing physical frailty, and to compare standard treatments for physical frailty. The results showed that both programs, when implemented independently, showed specific effects on subjective well-being, occupational performance, and physical fitness. However, QOL and physical fitness were significantly higher in the exercise group than in the literacy group. These results should be considered with caution because of the limited sample size of this pilot study.

## Figures and Tables

**Table 1:** Overall program flow of the literacy group and the exercise group.

	Literacy Group		Exercise Group
No.	The Theme of the Lecture	Group Work	Contents
Part 1	Program implementation, schedule confirmation, initial evaluation		
Part 2	Quality of life	Video on life performance and discussion on problem-solving	Exercises to prevent knee pain
Part 3	Life and Health	Discussion of learning topics	Exercise to prevent back pain
Part 4	Organization of space and objects	Video on life performance and discussion on problem-solving	Exercise to prevent falling over
Part 5	Review of progress on life goals and group work		Review of progress on life goals and group work, Exercises to prevent knee pain
Part 6	Adaptation of temporal organization and performance	Video on life performance and discussion on problem-solving	Exercise to prevent back pain
Part 7	Occupational analysis		Exercise to prevent falling over
Part 8	Completion Ceremony, Final Evaluation		

**Table 2:** Attributes of the final analysis subjects in both groups.

	Literacy Group (*n* = 13)	Exercise Group (*n* = 12)
Age (mean ± SD)	75.8 ± 4.76	76.5 ± 5.5
65–69	1	2
70–74	5	2
75–79	5	5
80–85	2	3
Gender		
Male	3	2
Female	10	10
Certified as requiring long-term care		
Support	3	0
Not applicable	10	12
Presence of disease (yes)	7	7
Presence of habits (yes)	8	8
Family living together (yes)	8	10
Frequency of going out (yes)	13	12

**Table 3:** Comparison of two factors for outcome measures.

		Literacy *n* = 13		Exercise *n* = 12		*p*-Value		
		Baseline ^1^	Post Intervention ^1^	Baseline ^1^	Post Intervention ^1^	Time	Group	Time × Group
TMIG-IC	Total	12.0 (1.6)	12.0 (1.4)	12.3 (1.2)	12.4 (0.9)	0.98	0.52	0.54
	Instrumental	4.9 (0.2)	5.0 (0)	4.9 (0.2)	4.8 (0.3)	0.95	0.37	0.17
	Active	3.6 (0.8)	3.7 (0.5)	3.8 (0.3)	3.9 (0.2)	0.28	0.39	0.74
	Social	3.5 (0.9)	3.2 (1.1)	3.5 (0.9)	3.6 (0.6)	0.50	0.48	0.24
LSI-Z	Total	8.4 (3.2)	8.6 (3.5)	8.5 (2.5)	9.5 (2.1)	0.03 *	0.69	0.11
WHOQOL26	Physical	23.1 (4.4)	23.1 (4.6)	26.4 (4.2)	26.1 (4.6)	0.84	0.07	0.84
	Psychological	19.9 (4.5)	19.3 (5.0)	21.4 (5.1)	22.5 (3.3)	0.66	0.19	0.12
	Social	9.3 (1.8)	9.8 (1.6)	11.0 (2.0)	11.2 (2.0)	0.19	0.04 *	0.69
	Environmental	25.5 (5.8)	26.6 (5.8)	30.9 (4.5)	30.9 (5.0)	0.39	0.02 *	0.39
GAS-L	Crude score	−1.0 (0)	0.3 (0.8)	−1.0 (0)	−0.1 (0.9)	0.01 **	0.19	0.19
Physical fitness tests	Grip strength	22.0 (5.5)	22.3 (6.4)	22.2 (6.0)	22.3 (6.0)	0.60	0.95	0.82
	Maximum 5 m	4.6 (2.3)	3.9 (1.4)	2.9 (1.1)	2.6 (0.7)	0.01 **	0.02 *	0.23
	TUG	9.8 (4.0)	8.9 (3.1)	7.5 (2.4)	6.0 (1.6)	0.01 **	0.03 *	0.20

* *p* < 0.05; ** *p* < 0.01; ^1^ Mean (S.D.); Instrumental—Instrumental independence; Active—Active intellectual activities; Social—Social role; Physical—Physical domain; Psychological—Psychological domain; Environmental—Environmental domain; Maximum 5 m—Maximum 5 m walking time.

## Data Availability

Data were not publicly deposited. However, we can share data and analyses that underpin the findings reported in this study. These are available on request from yu-ishi@tmu.ac.jp.
